# Practical and analytical considerations when performing interim analyses in diagnostic test accuracy studies

**DOI:** 10.1186/s41512-024-00174-4

**Published:** 2024-08-20

**Authors:** Susannah Fleming, Lazaro Mwandigha, Thomas R. Fanshawe

**Affiliations:** 1 Department of Primary Care Health Sciences, University of Oxford, Woodstock Road, Oxford, OX2 6GG UK; 2 GSK, GSK House, 980 Great West Road, Brentford, TW8 9GS UK

**Keywords:** Diagnostic accuracy, Interim analysis, Adaptive design, Group sequential methods, Stopping rule, Sensitivity, Specificity

## Abstract

Interim analysis is a common methodology in randomised clinical trials but has received less attention in studies of diagnostic test accuracy. In such studies, early termination for futility may be beneficial if early evidence indicates that a diagnostic test is unlikely to achieve a clinically useful level of diagnostic performance, as measured by the sensitivity and specificity. In this paper, we describe relevant practical and analytical considerations when planning and performing interim analysis in diagnostic accuracy studies, focusing on stopping rules for futility. We present an adaptation of the exact group sequential method for diagnostic testing, with R code provided for implementing this method in practice. The method is illustrated using two simulated data sets and data from a published diagnostic accuracy study for point-of-care testing for SARS-CoV-2. The considerations described in this paper can be used to guide decisions as to when an interim analysis in a diagnostic accuracy study is suitable and highlight areas for further methodological development.

## Introduction

The COVID-19 pandemic has highlighted the importance of rapid and accurate disease diagnosis to underpin treatment decisions and public health advice. While the practice of conducting diagnostic test accuracy (DTA) studies to estimate the performance of diagnostic tests, devices or decision rules is well-established [1], the pandemic brought into focus the need to assess new candidate diagnostics urgently to support their introduction into clinical practice.

Traditionally, prospective DTA studies use a single cohort design in which all participants receive one or more candidate diagnostic tests, with results compared against a reference standard, usually assumed to indicate the participant’s true disease status [2]. This design may be inefficient if an evaluation needs to be conducted at speed or if research resources may be more efficiently reallocated from a poorly-performing diagnostic test towards another test that may perform better. Programmes such as the National Institute for Health and Care Excellence (NICE) Early Value Assessment scheme demonstrate the increasing need for flexible designs that allow resources to be channelled rapidly towards technologies for which there is greatest need [3].

In these situations, it may be beneficial to consider interim analysis as part of DTA test accuracy study design. Particular importance may lie in the assessment of ‘futility’, allowing a DTA study to terminate if early indications suggest that the test is unlikely to reach a minimally acceptable diagnostic accuracy.

Interim analyses and adaptive trial design for DTA have received relatively little attention in the methodological literature, with papers by Gerke et al. and Zapf et al. being among the few papers to address this issue for studies outside the laboratory setting [4, 5]. In this paper, we provide an overview of how interim analysis methods can be applied to DTA studies and discuss practical considerations to guide decisions about performing such analyses. Generally, we assume that the objective is to assess the performance of a single diagnostic test (the ‘index test’) against a reference standard. In the context of DTA assessment, we demonstrate an implementation of an exact group sequential method-in which data are analysed at interim points after a certain number of participants have been recruited-and illustrate analytical issues using a study of a point-of-care diagnostic test for SARS-CoV-2 [6].

## Justification for interim analysis in DTA studies

Traditionally, most DTA studies have a target sample size based on either the total number of participants or the total number of disease cases, and are analysed and interpreted after this target has been reached. In many cases, this is an appropriate methodology and allows for clear justification of the sample size. Methods for determining fixed sample sizes for diagnostic accuracy studies are available elsewhere [7].

However, there are circumstances where it is appropriate to conduct interim analyses during data collection, by analogy with adaptive clinical trial design [8]. These can determine whether data collection should continue or if there is cause for early termination. Early termination may be appropriate if there is already sufficient evidence that the study is unlikely to yield a clinically useful result, known as termination for futility.

In the classic randomised controlled trial (RCT) design, termination for futility usually means that the intervention is unlikely to yield a statistically significant result or that if such a result were to be found, the effect size would be too small to be clinically important [9]. By reducing the number of ineffective treatment allocations, early termination for futility can make studies more efficient and cost-saving [10, 11]. Allowing stopping for either futility or efficacy may also considered to be ethical, as it prevents additional participants being exposed to the risks associated with additional tests or interventions [12].

In DTA study design, termination for futility may be similarly conceived as finding sufficient evidence that the test is unlikely to have clinically useful performance or to exceed minimum regulatory requirements. As DTA study results are typically expressed as a pair of summary measures-the sensitivity (true positive rate) and specificity (true negative rate)-the performance in relation to both measures should be considered when specifying a stopping rule.

A study may also be terminated early if there is sufficient interim evidence that it is very likely to yield a clinically useful result (termination for efficacy), although this practice has been criticised as likely to overestimate effect sizes [13]. In DTA studies, this would mean sufficient evidence that sensitivity or specificity is high enough to be clinically useful. This is less likely to be a reason to terminate a DTA study early, as continuing to the target sample size is rarely detrimental to the participants’ final diagnosis and would allow diagnostic performance to be estimated with greater precision. In most DTA studies, all participants receive the diagnostic test, so there is no subset of participants who might be considered to be disadvantaged by the study continuing, as might be the case in an RCT that used an inactive control.

A third possible reason for early termination, safety, is often based around consideration of adverse events [14]. In the case of DTA studies, this would require additional data from that used to estimate diagnostic performance, so this is not considered further here.

Early termination for futility or efficacy generally requires a stronger level of evidence that would be used at the planned end of the study, so as to be confident further data would be unlikely to change interpretation of the study results and to ensure the type I error rate is correctly controlled [8]. As for RCTs, when performing an interim analysis for a DTA study, it is advisable to pre-specify in the research protocol how many interim analyses will be conducted and their timing. If multiple interim analyses are planned, they do not need to be evenly spaced, and in DTA studies, the interim analysis points may be based on either the total number of participants recruited or the number of positive disease cases recruited.

## Practical considerations of interim analysis in DTA studies

Several practical considerations may influence the feasibility of carrying out an interim analysis for a DTA study. Researchers considering using an interim analysis in a DTA study should weigh up these practical aspects as well as the statistical points outlined in the subsequent sections

### Speed and availability of data collection

For DTA interim analysis to be feasible, both the index test and reference standard data must be available in a timely manner while the study is still going ahead. A time lag in obtaining index or reference data (e.g. from a laboratory) may result in additional participants being recruited to the study during the delay, reducing the potential benefit of the interim analysis. Planning of interim analyses should consider the expected speed of data flow.

### Blinding

If it is not possible to keep results of interim analysis hidden from individuals who recruit participants or perform the diagnostic or reference tests, consideration should be given to whether these assessments might be influenced by knowing the level of interim performance [4]. Loss of blinding may undermine the integrity of the DTA assessment [15].

### Timing of interim analyses

Timing of interim analyses should be chosen to reflect points where decisions about the continuation of the study can be made. The first interim analysis should not be planned before the sample size is sufficient to satisfy the assumptions of the chosen primary analysis.

### Accuracy of reference standard

In many DTA studies, the reference standard is imperfect. In some cases, a statistical adjustment can be made if an estimate of the accuracy of the reference standard is known using methods such as the Begg-Greenes adjustment [16]. Sometimes, an enhanced reference standard can be constructed by supplementing it with information from other sources, such as patient outcomes in long-term follow-up [17]. In the latter scenario, an interim analysis made on the basis of an imperfect reference standard may result in a different decision than one that would have been reached had the data required for the enhanced reference standard been available, and so an interim analysis may be less appropriate.

### Secondary outcomes

Typically interim analyses in DTA studies are based on the primary outcome of the sensitivity and/or specificity of the index test. Early termination reduces the potential to perform secondary analyses (e.g. on adverse events) and subgroup analyses for which the study may have lower power.

### Study resources

Interim analyses require additional work by the statistical team, which may need to be performed at speed if the study is recruiting rapidly. Thus, it is necessary to ensure that the study team is appropriately resourced to carry out any planned interim analyses.

### Cost of research

Carrying out interim analysis in a low-cost study with a low burden to study participants may not be an appropriate use of resources. However, if the study is expensive, difficult to recruit to, or has a high burden to participants, interim analyses have the potential to reduce costs and prevent further unnecessary data collection.

### Urgency of research

Interim analyses allow the time to implementation and potential patient benefit to be shortened, either by allowing accurate diagnostic tests to be introduced into practice more quickly or by advising against the use of poorly-performing tests.

### Impact on future research

A well-recognised limitation of interim analysis is the impact of early termination on systematic reviews and meta-analyses. Studies that have terminated early will contribute less data and will reduce the precision of pooled meta-analytic estimates. This must be balanced against the potential advantages of early termination.

## Adapting existing methods for interim analysis of DTA studies

In DTA studies, the primary analysis typically involves estimation of two proportions (sensitivity and specificity). Group sequential methods are one class of methods for interim analysis of binomial outcomes in RCTs that can be adapted for DTA studies, as described in this section.

### Exact group sequential method

Although DTA studies are often formulated in terms of being able to estimate sensitivity and/or specificity to acceptable precision (in terms of 95% confidence intervals), interim clinical trial methods can be adapted based on acceptance or rejection of a null hypothesis that represents a clinically important level of performance. For example, the sensitivity might be required to exceed a given level for the test to be considered suitable for adoption into practice.

As the proportion to be tested in RCTs is typically small, some methods, including the exact group sequential method, rely on an assumption that this proportion is less than 0.5 [18, 19]. However, desired termination values for sensitivity and specificity are likely to be greater than 50%. We therefore recommend using these methods on the false negative rate (FNR, 1-sensitivity) and the false positive rate (FPR, 1-specificity) rather than directly on the sensitivity and specificity.

An example of a null hypothesis for DTA study might be ‘FNR $\le$ 15%’, equivalent to ‘sensitivity $\ge$ 85%’. In general,

$$\begin{aligned} H_0: p \le p_t \end{aligned}$$

where *p* is the true FNR, and $p_t$ is the ‘threshold proportion’, in this case 0.15. We also define $p_0=1-p_t$ as the corresponding threshold in terms of sensitivity or specificity. Our alternative hypothesis is

$$\begin{aligned} H_1: p > p_t. \end{aligned}$$

Stopping rules are also affected by $\alpha$, the probability of type I error (i.e. incorrectly rejecting the null hypothesis). In this formulation, rejection of the null hypothesis in a DTA study corresponds to stopping for futility, which is the most likely practical application of interim analysis in this context.

**Fig. 1:**
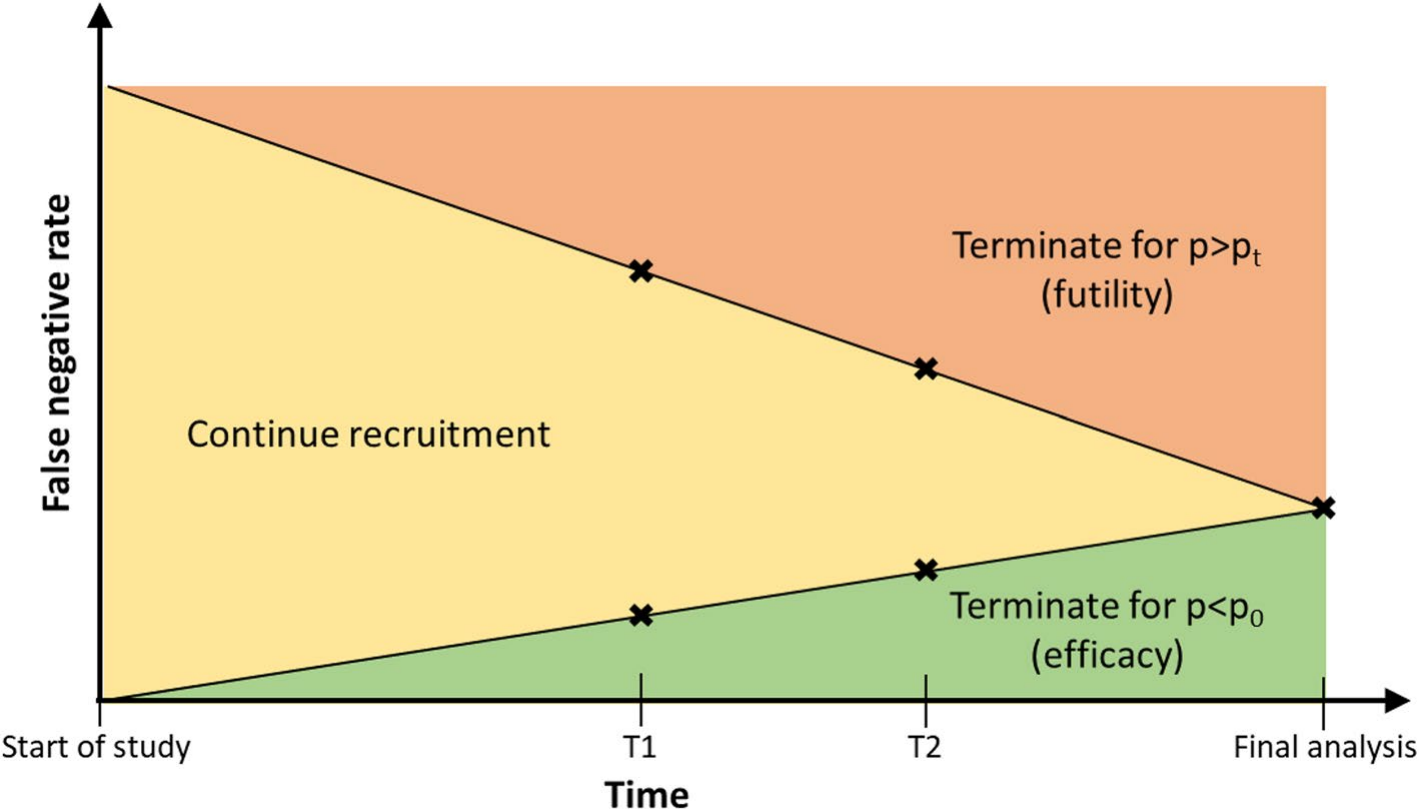
Schematic illustration of boundaries used for early termination for false negative rate (1-sensitivity), with two pre-defined interim analyses. In this example, there are two interim analysis points, at *T*1 and *T*2. The crosses mark the thresholds for stopping for efficacy and futility. Termination for false positive rate would follow a similar pattern. The boundaries are shown as straight lines for simplicity, but this need not be the case

Group sequential methods define two sets of ‘boundaries’, or ‘thresholds’, that are used to determine whether early stopping is appropriate [20]. Figure 1 demonstrates this graphically. The boundaries calculated by the exact group sequential method are fixed for any given planned sample size. It is recommended that the number of interim analyses using the exact group sequential method should not be greater than five, to prevent excessive risk of type I error [18, 21].

We have implemented the ‘exact group sequential’ method [18, 19] in R, incorporating adjustments to apply to DTA studies. Appendix A describes the exact group sequential method in more detail.

## Example implementations

### Simulated data

**Fig. 2:**
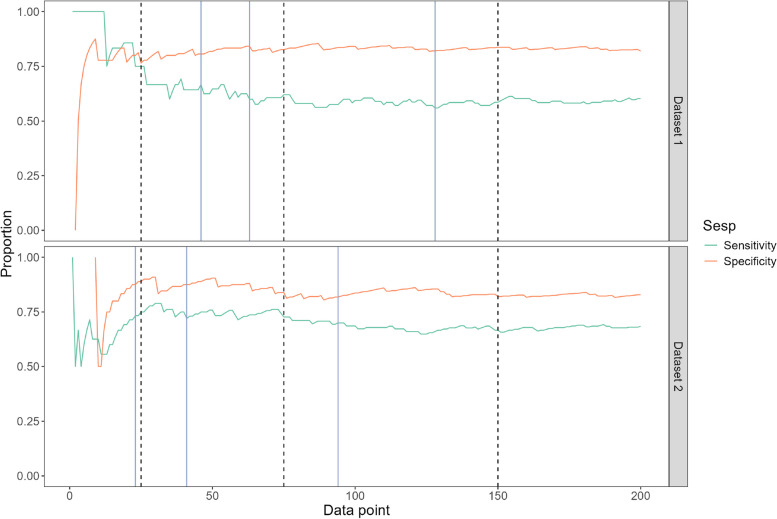
Continuously-estimated sensitivity and specificity for the example datasets, plotted against the number of participants recruited. Dashed black vertical lines show the positions of the interim analysis points after a total of 25, 75 and 150 participants. Solid blue vertical lines show the position of the interim analysis points after 15, 25 and 50 disease-positive cases

We illustrate use of the exact group sequential method using two artificial datasets, randomly generated to simulate a DTA study where the true sensitivity in the underlying population is 65%, with specificity 85% and prevalence 35%. Figure 2 shows estimated sensitivity and specificity as recruitment accrues. Further details of the datasets and the corresponding R code are provided in Appendix B.

**Fig. 3:**
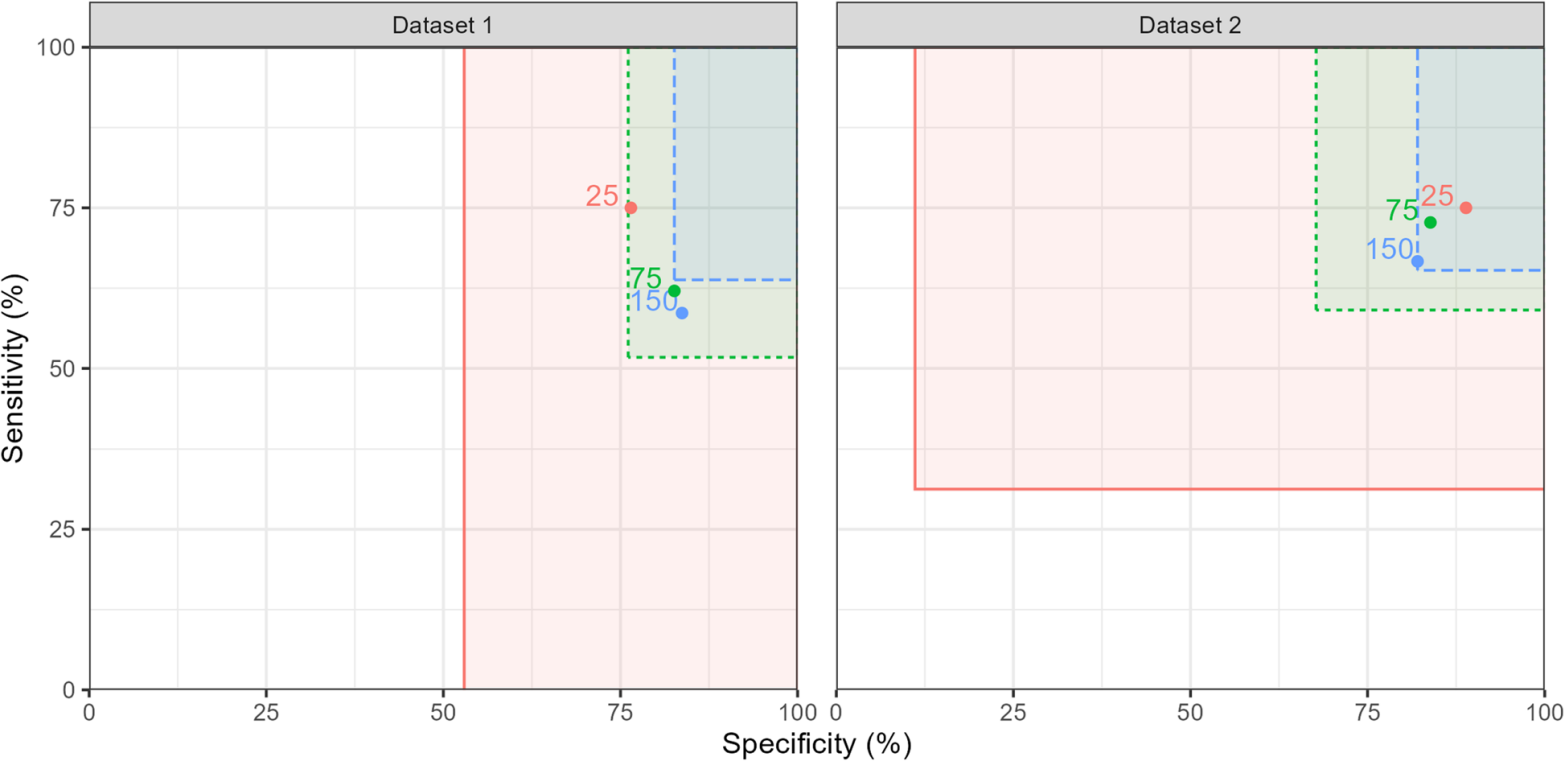
Rectangles representing the sensitivity and specificity boundaries for termination for futility at interim analysis points after 25 (red), 75 (green) and 150 (blue) participants for the example datasets. Labelled points show the estimated sensitivity and specificity at these interim analyses

Figure 3 shows the sensitivity and specificity at three proposed interim analysis points: after 25, 75 and 150 participants have been recruited, with a target total sample size of 200. The rectangles represent the sensitivity and specificity boundaries for termination for futility, with $p_0$ set as 75% for sensitivity and 90% for specificity. We have not considered termination for efficacy, since we believe that would rarely be appropriate in a DTA study. Where the estimate falls within the box, termination will not be advised. Table 1 shows this data in numerical form. As the figure and table show, neither early termination was not indicated for either dataset at $n=25$ or $n=75$, even though some of the sensitivity and specificity estimates fell below $p_0$ at these points. At $n=150$, dataset 1 indicated termination for futility in sensitivity, and dataset 2 borderline termination for futility in specificity.

**Table 1:** Specificity and sensitivity estimates, and termination boundaries, for the example datasets with interim analyses after 25, 75, and 150 participants, and with $p_0$ set to 75% for sensitivity, and 90% for specificity. Interim analysis points are defined by *N*, the total number of participants recruited and $N_{pos}$ the number of disease-positive cases observed at the corresponding point. Boundaries below 0 are show as dashes

	*N* ($N_{pos}$)	Sensitivity (boundary)	Specificity (boundary)
Dataset 1	25 (8)	75% (-)	76% (53%)
		Continue	Continue
	75 (29)	62% (52%)	83% (76%)
		Continue	Continue
	150 (58)	59% (64%)	84% (83%)
		Stop for futility	Continue
Dataset 2	25 (16)	75% (31%)	89% (11%)
		Continue	Continue
	75 (44)	73% (59%)	84% (68%)
		Continue	Continue
	150 (72)	67% (65%)	82% (82%)
		Continue	Stop for futility

**Fig. 4:**
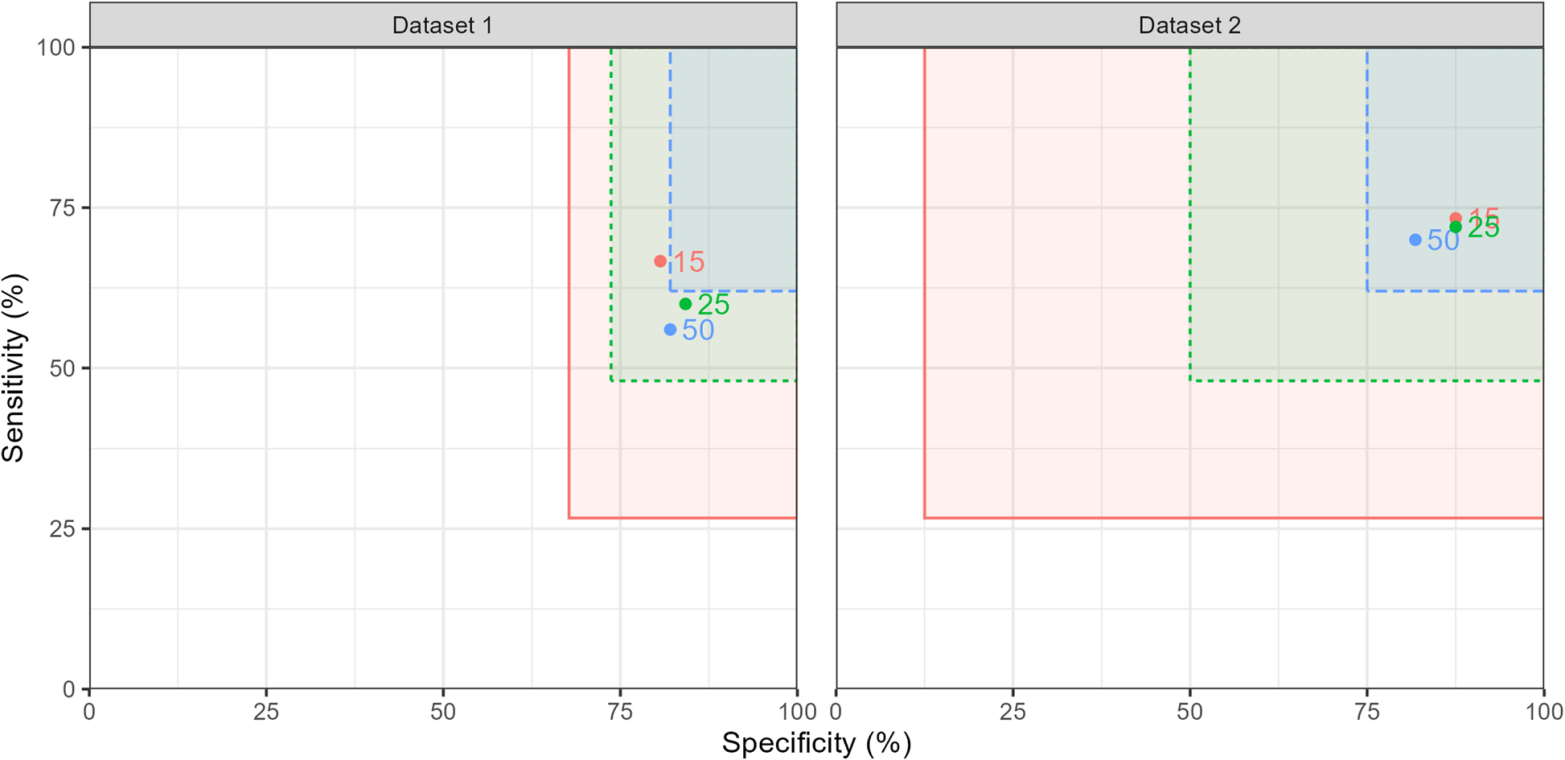
Rectangles representing the sensitivity and specificity boundaries for termination for futility at interim analysis points after 15 (red), 25 (green) and 50 (blue) disease-positive cases for the example datasets. Labelled points show the estimated sensitivity and specificity at these interim analyses

Interim analysis points can also be defined in terms of the number of disease-positive participants recruited, using projected numbers of disease-negative participants for the specificity interim analysis at the same points. Figure 4 and Table 2 show the same data for interim analyses after 15, 25 and 50 disease-positive cases. In this scenario, dataset 2 does not meet the termination thresholds at any interim point assessed.

**Table 2:** Specificity and sensitivity estimates, and termination boundaries, for the example datasets with interim analyses after 15, 25 and 50 disease-positive cases, and with $p_0$ set to 75% for sensitivity, and 90% for specificity. Interim analysis points are defined by $N_{pos}$, the number of disease-positive cases recruited, and *N* is the total number of participants recruited at the corresponding point

	*N* ($N_{pos}$)	Sensitivity (boundary)	Specificity (boundary)
Dataset 1	46 (15)	67% (27%)	81% (68%)
		Continue	Continue
	63 (25)	60% (48%)	84% (74%)
		Continue	Continue
	128 (50)	56% (62%)	82% (82%)
		Stop for futility	Stop for futility
Dataset 2	23 (15)	73% (27%)	88% (12%)
		Continue	Continue
	41 (25)	72% (48%)	88% (50%)
		Continue	Continue
	94 (50)	70% (62%)	82% (75%)
		Continue	Continue

Figures 3 and 4 illustrate how the rectangles defined by the termination boundaries shrink as the sample size increases. The sensitivity boundaries in Fig. 4 for the two datasets match, as the number of disease-positive cases are equal in this scenario.

### Case study: RAPTOR-C19

RAPTOR-C19 is a platform DTA study assessing point-of-care tests for SARS-CoV-2 against a reference standard PCR test. We use as an example the first two tests (‘SD Biosensor’ and ‘BD Veritor’) assessed by this study [6], to illustrate the use of the group sequential method in different scenarios. In this case study, we assume that interim analyses were planned after 50, 100 and 150 COVID-19 cases had been observed although the available interim points slightly exceeded these numbers as data were only available daily and several participants were usually recruited each day (see Appendix C for raw data). Therefore, the first interim analysis after 50 positive cases actually includes 52 positive cases for the BD Veritor device, and 53 for the SD Biosensor device, and the second interim analysis after 100 positive cases actually includes 103 positive cases for both devices. We used the original target sample size of 150 COVID-19 cases, with an assumed prevalence of 30%, to determine the expected sample sizes for sensitivity and specificity.

**Table 3:** MHRA target product profiles, used to define $p_0$ for the RAPTOR case study

	Sensitivity	Specificity
Desirable	97%	99%
Acceptable	80%	95%

**Fig. 5:**
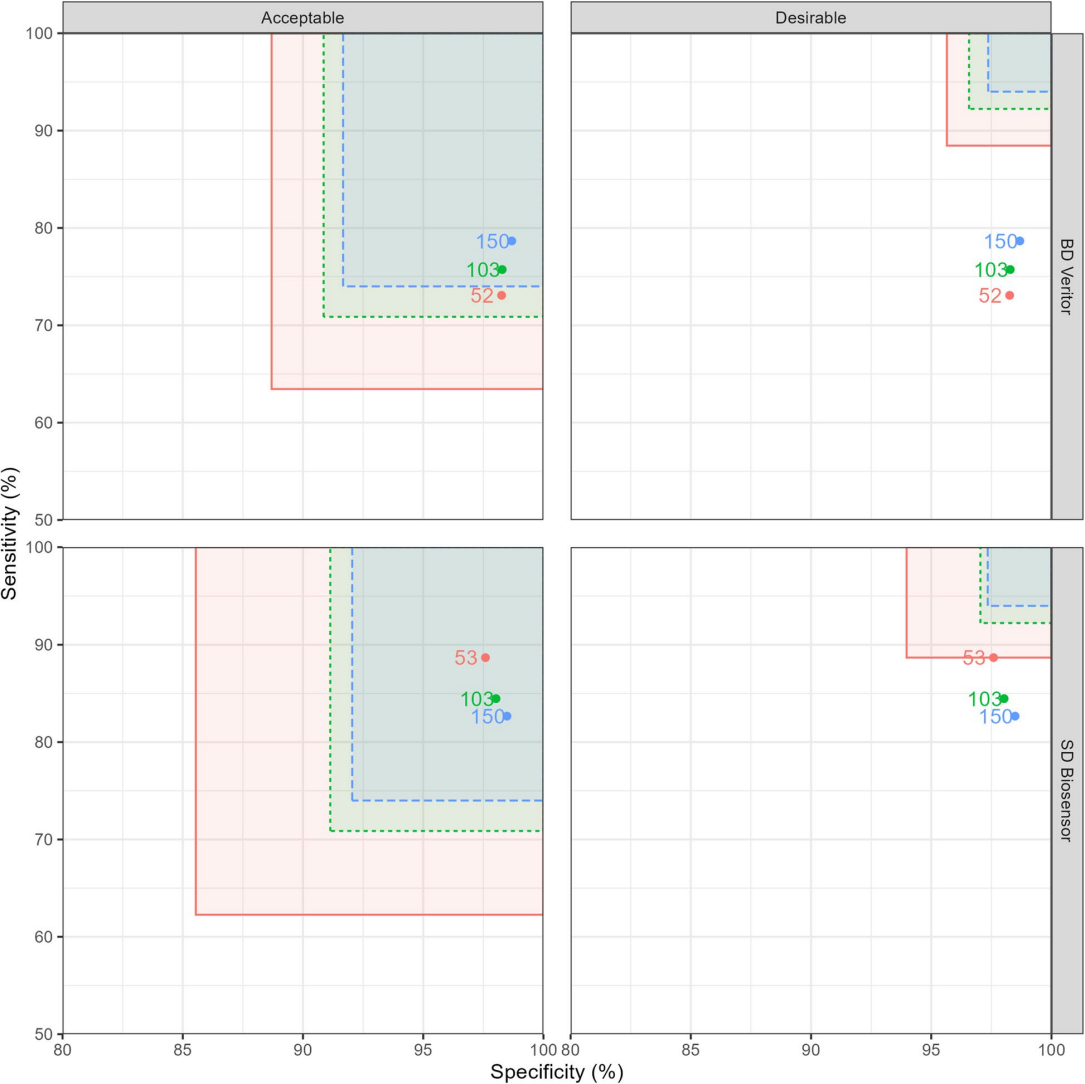
Rectangles representing the sensitivity and specificity boundaries for termination for futility for two point-of-care tests at interim analysis points as soon as possible after 50 (red), 100 (green) and 150 (blue) COVID-19 cases in the RAPTOR-C19 study. Labelled points show the estimated sensitivity and specificity at these interim analyses

We assume here that stopping for futility may occur if either sensitivity or specificity meets the stopping criterion and do not consider stopping for efficacy. We test two specifications of $p_0$ for illustration, as defined by the Medicines & Healthcare products Regulatory Agency (MHRA) Target Product Profiles (Table 3). In a real DTA study, the choice of threshold specification would have to be made a priori and documented in the study protocol. Figure 5 shows the thresholds for each point-of-care test at the two different product profiles, and Table 4 shows the decisions for each option.

**Table 4:** Termination points for the RAPTOR-C19 case studies. $N_{pos}$ denotes the number of COVID-19 cases at the interim analysis. Boundary values for sensitivity and specificity at the interim analysis are shown in brackets to allow direct comparison with the observed values

Device	Product profile	Interim analysis	Observed sensitivity (boundary)	Observed specificity (boundary)	Decision
BD Veritor	Desirable	1 ($N_{pos}=52$)	73.1% (88.5%)	98.3% (95.7%)	Stop for futility
BD Veritor	Acceptable	3 ($N_{pos}=150$)	78.7% (74.0%)	98.7% (91.7%)	No termination
SD Biosensor	Desirable	1 ($N_{pos}=53$)	88.7% (88.7%)	97.6% (94.0%)	Stop for futility
SD Biosensor	Acceptable	3 ($N_{pos}=150$)	82.7% (74.0%)	98.5% (92.0%)	No termination

In Fig. 5 and Table 4, stopping points are not reached for either device under the ‘acceptable’ product profile limit, and so the final interim analysis occurs after 150 positive cases. Under the more stringent ‘desirable’ specification, termination would have occurred at the first interim analysis for both devices (after 50 positive cases), due to low sensitivity.

## Other statistical considerations when conducting interim analyses in DTA studies

Other statistical considerations may mean different analytical approaches may be suitable in some circumstances, as outlined below.

### Incorporating both sensitivity and specificity

DTA studies are unusual in having a bivariate sensitivity and specificity outcome. Although these were considered independently in the previous section, they might also be modelled jointly with the error rate adapted for a bivariate response [22–24].

For termination for efficacy, we advise that termination thresholds for both sensitivity and specificity should be met before termination occurs. In contrast, in some circumstances, termination for futility in DTA studies may be appropriate if the threshold for either sensitivity or specificity is met, as in the example above where a test might be required to meet a minimum performance level on both measures.

### Other outcome measures

This paper focuses on the use of sensitivity and specificity as co-primary endpoints. Group sequential methods can also be adapted for other outcome measures, such as those based on the receiver operating characteristic curve, if the index test does not give a binary result. In these situations, a suitable outcome may be the area under the curve [25, 26] or the detection of a point on the curve that exceeds a minimum sensitivity or specificity. The methods described in this paper could be used for positive and negative predictive values, as these are also proportion measures. The method could be further adapted for other outcomes such as the diagnostic odds ratio or likelihood ratios.

### Bias and precision of parameter estimates

Most interim analysis methods are based on hypothesis testing and the need to preserve type I error rates. Often in DTA studies the precision of the sensitivity and specificity estimates is more important than a *p*-value from a hypothesis test. Unadjusted parameter estimates from studies that terminate early for futility are known to be biased and therefore a bias-correction is required [27–29]. Estimates resulting from a study that has terminated early for futility will also be less precise than those from a study that has progressed to the target sample size.

### Discrete or continuous interim analysis

The exact group sequential approach outlined above is suitable for situations in which interim analysis is to be carried out at up to five points. In some situations, it may be possible to conduct an ongoing sequential procedure in which performance is continuously assessed as each data point arrives, although as previously noted, there may be practical constraints when attempting this in the DTA context. In these scenarios, an adaptation of the alpha ‘spending function’ approach may be considered [30, 31].

### Sample size re-estimation

Sample size estimates for DTA studies often require an estimate of the anticipated prevalence of the outcome. An alternative use of interim analysis is therefore to check whether the observed prevalence is close to that originally assumed and if necessary re-estimate the required sample size while the study is ongoing. This practice has been reviewed both generally [32] and applied to DTA studies [33, 34].

### Multiple index tests

Platform DTA studies in which more than one test is evaluated concurrently are becoming increasingly common. If multiple diagnostic tests are performed in parallel, interim analysis methods could be adapted to eliminate the worse-performing tests as the study proceeds, using methods similar to ‘drop-the-loser’ adaptive clinical trial designs [35, 36].

## Discussion

This paper has described practical and analytical considerations that should be considered before undertaking interim analysis of a DTA study. This research area remains underdeveloped, and there are further challenges in harmonising existing research from the traditional adaptive design literature with diagnostic accuracy methodology.

A strength of our work is that it is one of few papers to have directly addressed the issue of interim analysis in DTA studies. It provides practical advice about considerations that should be made and illustrates analytical work with case studies. A limitation is that it does not attempt to implement all of the many interim analysis methods that have been proposed in the clinical trial literature.

The paper also highlights some novel challenges that are specific to DTA research, including use of sensitivity and specificity as a bivariate outcome, as a composite primary outcome measure. Uniquely in DTA studies, the total ‘n’ which contributes to each of these outcomes cannot always be fixed in advance unless a case-control design, not generally recommended for DTA studies, is used; this creates analytical complexity when using existing methods. An important future research direction is the use of multiple diagnostic index tests concurrently, as might be adopted in ‘drop-the-loser’-type designs. This design appears likely to grow in importance as the rate at which new point-of-care diagnostics are developed increases.

## Data Availability

The datasets and code generated and analysed in this paper are available on Github (https://github.com/OxPrimaryCareStats/DTA-interim-analysis). Readers are welcome to use this code to implement the methods described in this paper, but should be aware that the code is provided ‘as is’, and no guarantee is given as to its accuracy. All code is created and designed for use in R [37] and has been run in R version 4.2.2. Comments are provided in each file to describe the usage and effects of the code.

## A The exact group sequential method

We have implemented the exact group sequential method described by Zhao [19] and based on the method of Fleming [18] with adjustments for DTA studies. The R code for implementing the methods described in this section can be found in DTAinterimAnalysis.R.

In this method, at each interim analysis point *g*, acceptance ($a_g$) and rejection ($r_g$), thresholds are determined based on binomial probabilities, as specified in Eqs. 1 and 2. Decisions are based on the number of false positive or false negative events, $s_g$, observed at the interim analysis point.

- If $s_g \le a_g$ then $H_0$ is accepted on the basis of efficacy, or the study continues if a futility-only assessment if being performed.
- If $s_g \ge r_g$ then $H_0$ is rejected on the basis of futility.
- If $a_g< s_g < r_g$ then the study continues until the next interim analysis point.

1 $$\begin{aligned} a_g = \left[ \sum \limits ^g_{i=1} n_g p_A - z_{1-\alpha } \left\{ N p_A (1-p_A) \right\} ^{\frac{1}{2}} \right] ^* \end{aligned}$$

2 $$\begin{aligned} r_g = \left[ \sum \limits ^g_{i=1} n_g p_t + z_{1-\alpha } \left\{ N p_t (1-p_t) \right\} ^{\frac{1}{2}} \right] ^* +1 \end{aligned}$$

Where

$$\begin{aligned} p_A = \frac{(N p_t)^{\frac{1}{2}} + (1-p_t)^{\frac{1}{2}} (z_{1-\alpha })^2 }{N + (z_{1-\alpha })^2 } \end{aligned}$$

$a_g$ and $r_g$ are dependent on four variables:

$n_g$: The number of data points up to the interim analysis
*N*: The proposed final sample size of the study
$\alpha$: The probability of Type I error
$p_t$: The threshold proportion of events, chosen so that $H_0: p \le p_t$.

In Zhao [19] and Fleming [18], the final analysis point will always result in either acceptance or rejection of $H_0$, since $a_g=r_g-1$ is substituted for Eq. 1 at the final analysis point. However, this is not implemented in our code, as final conclusions in DTA studies are typically based on confidence intervals for sensitivity and specificity rather than solely the acceptance or rejection of a null hypothesis concerning either measure.

In the case of DTA interim analysis, the direction of $H_0$ requires *p* and $p_t$ to be defined in terms of the false positive rate or the false negative rate. The code carries out the conversion from sensitivity and specificity (and hence $p_0$), so that the user does not have to deal in terms of the false positive rate or the false negative rate, but internal calculations use these values.

## B Example data and code

### Example datasets

The example datasets used in this paper can be generated using the R script createTestData.R. This creates two example datasets (Fig. 2) with the same basic characteristics, but different individual patterns of data points. The datasets are created with 1000 data points and nominal sensitivity of 65%, specificity of 85% and prevalence of 35%. For the analyses and testing described in this paper, the first 200 data points of each dataset were used to simulate a realistic DTA study.

### Implementing interim analysis for DTA studies

The two main functions provided to implement DTA interim analysis using the exact group sequential method are DTAdiscreteInterimAnalysis() and DTAcumulativeInterimAnalysis(). Both functions are provided in DTAinterimAnalysis.R and their use is demonstrated in DTAexampleCode.R. The choice of function is determined by the form of the data to be analysed.

If the data can easily be converted to paired logical (true/false) results for the reference and index tests, in the order that data were collected, then DTAdiscreteInterimAnalysis() can be used. This takes as an input a data frame containing, as a minimum, columns of logical data named reference (containing the results for the reference test), TP (whether the test was a true positive), and TN (whether the test was a true negative). A helper function, continuousSeSp() , is provided in generateDTAdata.R, which can add these and other useful columns to a data frame containing logical columns for the reference and index tests. This function also takes an argument specifying at which points interim analysis should be carried out.

In some DTA studies, it will be easier to provide a snapshot of the data at the desired interim analysis points. This sort of data is handled by DTAcumulativeInterimAnalysis(). This takes a data frame with four columns as an input: N (the number of data points included in the interim analysis), RefT (the number of positive reference test results up to the interim analysis point), TP (the number of true positives up to the interim analysis point) and TN (the number of true negatives up to the interim analysis point).

The inputs to these functions are:

pSe: The desired threshold for sensitivity (as a proportion on the scale 0–1)
pSp: The desired threshold for specificity (as a proportion on the scale 0–1)
prevalence: The expected prevalence for the study
N: The planned total sample size (only one of N or Positive N should be provided, depending on the sample size calculation)
PositiveN: The planned number of positive cases (only one of N or Positive N should be provided, depending on the sample size calculation)
alpha: The acceptable one sided nominal type I error (defaults to 0.05)
simpleOutput: binary variable determining whether a simplified or detailed output is provided (defaults to true, giving the simplified output)

As the interim analysis is carried out separately for sensitivity and specificity, it is necessary to know the planned number of disease-positive and disease-negative cases, as defined by the expected prevalence and either the planned total sample size, or the planned number of cases. However, it is possible that the actual number of either disease-positive or disease-negative cases may exceed this, either due to chance variation or because the expected prevalence was incorrect. If the number of actual cases at any interim point exceeds the planned number, the code will inflate the planned number to accommodate this. The code will warn the user that the number has been inflated but will continue to produce results. It should be noted that the planned number is inflated for all analyses.

Other functions and files exist in the GitHub repository. These are typically ‘helper’ functions or were created to support the analysis underlying this paper. Comments are provided above the function description, which should assist in explaining their use.

## C RAPTOR-C19 interim analysis data

Table 5 shows the data from the RAPTOR-C19 trial, which was used to carry out the interim analyses in Table 4 and Fig. 5. In the full study, recruitment continued for a short period after the desired number of cases (150) was obtained, but in the case study, we have used data only up to when 150 cases were recruited.

**Table 5:** Data on performance of the two test devices at interim analysis points for the RAPTOR-C19 case study

Device	$N_{pos}$	Total *N*	True positives	True negatives
BD Veritor	52	167	38	113
BD Veritor	103	278	78	172
BD Veritor	150	378	118	225
SD Biosensor	53	136	47	81
SD Biosensor	103	306	87	199
SD Biosensor	150	414	124	260
